# Complementary and Alternative Medicine for Diabetes

**DOI:** 10.1155/2013/831068

**Published:** 2013-10-03

**Authors:** Wen-Chin Yang, Srinivas Nammi, Per Bendix Jeppesen, William C. S. Cho

**Affiliations:** ^1^Agricultural Biotechnology Research Center, Academia Sinica, Taipei 11501, Taiwan; ^2^School of Science and Health, University of Western Sydney, NSW 2751, Australia; ^3^Department of Endocrinology and Metabolism, Aarhus Sygehus THG, Aarhus University Hospital, Tage-Hansens Gade 2, 8000 Aarhus C, Denmark; ^4^Department of Clinical Oncology, Queen Elizabeth Hospital, Hong Kong

Although diabetes was identified by a Greek physician, Aretaeus of Cappadocia, about 2,000 years ago, this old disease remains incurable. Diabetes is characterized by insulin deficiency, insulin resistance, and aberrant glucose, protein, and lipid metabolism. Genetic and environmental factors are the primary causes of diabetes. It is estimated that about 300 million people globally are afflicted with this disease. However, current oral antidiabetic agents using orthodox medicine have unmet efficacy and undesirable side effects in patients, leading to the development of microvascular and macrovascular complications. Research and development of new remedies for diabetes are, therefore, in great demand.

It is estimated that complementary and alternative medicine (CAM) is used by 80% of the world population for primary health care. Therefore CAM, including herbal medicines, acupuncture, moxibustion, and other therapies, represents an important area of exploration for diabetes therapy. In this special issue, we aimed to gather together updated information reflecting the considerable progress in basic and clinical research into CAM for diabetes and its complications.

The papers in this special issue cover a wide range of topics, including *in vitro* studies, preclinical studies, and clinical trials on CAM for diabetes and related diseases. One *in vitro* study by S.-C. Chang and W.-C. V. Yang titled “*Hyperglycemia induces altered expressions of angiogenesis associated molecules in the trophoblast*” describes the expression of perlecan and angiogenesis-related cytokines and growth factors in trophoblasts, one type of placenta cells. High glucose affected the expression level of cell-bound perlecan, angiogenesis-associated cytokines, and the matrix degradation on the cells, implying that hyperglycemia influences vessel formation during placentation. A. Nachar et al. (“*The action of antidiabetic plants of the Canadian James Bay Cree traditional pharmacopeia on key enzymes of hepatic glucose homeostasis*”) report the antidiabetic action of seven Canadian plants as evidenced by glucose-6-phosphatase and glycogen synthase, two key enzymes, respectively, involved in gluconeogenesis and glycogenesis in hepatocytes. Among them, *Abies balsamifera* and *Picea glauca* decreased glucose-6-phosphatase activity. This decrease involved the Akt and AMPK pathways. In contrast, *Larix laricina* and *A. balsamifera* increased glycogen synthase activity. Preclinical studies were used to study the action and mechanism of CAM in rodents. Diabetes arises from a defect in *β* cell functions and insulin resistance. A small flavone-type molecule, swertisin, found in *Enicostemma littorale* was tested for its ability to promote the generation of pancreatic islets. N. Dadheech et al. (“*A small molecule swertisin from Enicostemma littorale differentiates NIH3T3 cells into islet-like clusters and restores normoglycemia upon transplantation in diabetic Balb/c mice*”) showed that swertisin could promote the differentiation of NIH3T3 cells into pancreatic islet-like cell mass, which restored hypoglycemic status to normal. Another study by C. L.-T Chang et al. (“*Antidiabetic effect and mode of action of cytopiloyne*”) reported that a small molecule, cytopiloyne, isolated from *Bidens pilosa*, improved type 2 diabetes (T2DM) in db/db mice via its modulation of *β*-cell functions (insulin production and *β*-cell preservation) involving the calcium/DAG/PKC*α* cascade. J. Wang and colleagues (“*Improvement of liquid fructose-induced adipose tissue insulin resistance by ginger treatment in rats is associated with suppression of adipose macrophage-related proinflammatory cytokines*”) show that treatment with ginger extract reduced fructose-induced insulin resistance in rats by suppression of adipose inflammatory cytokines (TNF-*α*, IL-6, MPC-1, CCR-2, etc.) and increased phosphorylation of IRS-2. K. K. Tan and K. H. Kim. (“*Alternanthera sessilis red ethyl acetate fraction exhibits antidiabetic potential on obese type 2 diabetic rats*”) report that, despite their inability to identify active compounds, they observed that ethyl acetate fraction of *Alternanthera sessilis* ameliorated T2D via increased insulin content and decreased insulin resistance. Aside from blood glucose, this fraction reduced blood triglyceride and free fatty acids. S. H. Kim and coworkers (“*Citrus junos Tanaka peel extract exerts antidiabetic effects via AMPK and PPAR-γ both in vitro and in vivo in mice fed a high-fat diet*”) indicate that the ethanol extract of the peel of *Citrus junos in vitro *stimulates glucose uptake in C2C12 myotube cells. This extract also augmented activity of PPAR-*γ* and AMPK in C2C12 cells. Consistent with the *in vitro* data, the extract diminished insulin resistance as well as body weight and adipokines and elevated AMPK phosphorylation in high-fat diet- (HFD-) induced mice. N. Hu et al. (“*Anti-diabetic activities of Jiaotaiwan in db/db mice by augmentation of AMPK protein activity and upregulation of GLUT4 expression*”) demonstrate the antidiabetic action of a traditional Chinese medicine (TCM), Jiaotaiwan, as shown by reduction of blood glucose level and enhancement of islet protection, hepatic AMPK activity, and expression level of glucose transporter 4 in skeletal muscle and white fat. H.-Y. Huang and colleagues (“*Supplementation of Lactobacillus plantarum K68 and fruit-vegetable ferment along with high fat-fructose diet attenuates metabolic syndrome in rats with insulin resistance*”) report that a mixture of fruit/vegetable ferment and one of its bacteria, *Lactobacillus plantarum* K68, reduced hyperglycemia, hyperinsulinemia, and hyperlipidemia as well as proinflammatory cytokines (TNF-*α*, IL-6, IL-1*β*, etc.) in HFD-induced rats. This reduction was associated with a decrease in insulin resistance. P. V. Rao and coworkers (“*Rhinacanthus nasutus ameliorates cytosolic and mitochondrial enzyme levels in streptozotocin-induced diabetic rats*”) showed that glycolytic enzymes such as glucose-6-phosphate dehydrogenase, succinate dehydrogenase, glutamate dehydrogenase, and lactate dehydrogenase were upregulated in diabetic mice. In contrast, the methanol extract of *R. nasutus* reduced those enzymes, implying that this extract exerts antidiabetic action via reduction of metabolic enzymes. In addition, two articles delineate the impact of herbal medicine and compounds on diabetic complications in animals (“*Proanthocyanidin attenuation of oxidative stress and NF-κB protects apolipoprotein E-deficient mice against diabetic nephropathy*” and “*An aqueous extract of Radix Astragali, Angelica sinensis, and Panax notoginseng is effective in preventing diabetic retinopathy*”). One study by D. Gao and colleagues (“*An aqueous extract of Radix Astragali, Angelica sinensis, and Panax notoginseng is effective in preventing diabetic retinopathy*”) reports that Dang Gui Bu Xue Tang, a TCM composed of *A. membranaceus*, *A. sinensis*, and *P. notoginseng*, reduced diabetic retinopathy in diabetic Goto-Kakizaki rats and/or streptozotocin- (STZ-) induced rats. This reduction was associated with retinal downregulation of proinflammatory cytokines and the reversal of glucose-induced inhibition of endothelial cell migration/proliferation *in vitro*. Another study shows that treatment with the polyphenolic compounds proanthocyanidins reduced nephropathy in STZ-treated apolipoprotein E-deficient mice (“*Proanthocyanidin attenuation of oxidative stress and NF-κB protects apolipoprotein E-deficient mice against diabetic nephropathy*”). This reduction is relevant to its attenuation of oxidative stress and NF-*κ*B activation.

In human clinical studies, X. Tu et al. (“*Fructus mume formula in the treatment of type 2 diabetes mellitus: a randomized controlled pilot trial*”) demonstrate that 12-week treatment with a monofactorial formula, *F. mume*, reduced blood glucose in 41 patients of T2DM. X. Li and colleagues (“*The rs1142345 in TPMT affects the therapeutic effect of traditional hypoglycemic herbs in prediabetes*”) concluded that a single nucleotide polymorphism (rs1142345) in thiopurine *S*-methyltransferase could affect the therapeutic outcome of the patients with T2DM receiving the TCM, Tianqi Jiang Tang. C. X. Huang et al. (“*Prescription pattern of Chinese herbal products for diabetes mellitus in Taiwan: a population-based study*”) present the use and likely mechanisms of action of multifactorial formulae such as Liu-Wei-Di-Huang-Wan and its derivatives. Their antidiabetic mechanisms are multifaceted and include increase in insulin secretion, insulin sensitivity, peripheral glucose uptake, diminution of intestinal glucose absorption, hepatic glucose, production and insulin resistance. Despite some evidence in favor of their use, the data could be confounded by the placebo effect, suggesting that well-conducted, double-blind, randomized, placebo-controlled studies are required for further investigations. Further, C. I. Tsai et al. (“*Chinese medicinal formula (MHGWT) for relieving diabetic neuropathic pain: a randomized, double-blind, placebo-controlled trial*”) investigated the therapeutic effect of a modified TCM formula, Hungqi Guizhi Wuwu Tang, on neuropathic pain in 112 diabetic patients, as assessed by 15-item short-form brief pain inventory and the 17-item short-form McGill pain questionnaire. This TCM significantly improved neuropathic pain in the patients.

In addition to original research articles, this special issue also features review articles. One review entitled “*Herbal therapies for type 2 diabetes mellitus: chemistry, biology, and potential application of selected plants and compounds*” describes a variety of antidiabetic herbal products from chemical, biological, pharmacological, and clinical aspects. And another review entitled “*Adjunct methods of the standard diabetic foot ulceration therapy*” summarizes three adjunct therapies, hyperbaric oxygen therapy, maggot therapy, and platelet-rich plasma therapy in one of the most serious diabetic complications, foot ulcers. The authors discuss preclinical and clinical studies of those therapies together with their effects and modes of action in animals and humans. Both review articles shed light on further directions for CAM in diabetes and diabetic complications.

We envision that this special issue will attract broad interest in the field of diabetes and encourage the perusal of more in-depth investigations into the use of CAM-based therapies for diabetes and the related complications.

*Wen-Chin Yang* *Wen-Chin Yang* *Srinivas Nammi* *Srinivas Nammi* *Per Bendix Jeppesen* *Per Bendix Jeppesen* *William C. S. Cho* *William C. S. Cho*

